# 2,4,6,8-Tetra­kis(4-bromo­phen­yl)-3,7-diaza­bicyclo­[3.3.1]nonan-9-one

**DOI:** 10.1107/S1600536809055226

**Published:** 2010-01-09

**Authors:** Wan-Sin Loh, Hoong-Kun Fun, S. Sarveswari, V. Vijayakumar, B. Palakshi Reddy

**Affiliations:** a X-ray Crystallography Unit, School of Physics, Universiti Sains Malaysia, 11800 USM, Penang, Malaysia; b Organic Chemistry Division, School of Advanced Sciences, VIT University, Vellore 632 014, India

## Abstract

In the title compound, C_31_H_24_Br_4_N_2_O, one of the bromo­phenyl rings is disordered over two orientations with occupancies of 0.69 (2) and 0.31 (2). The bicyclo­[3.3.1]nonane ring system adopts a chair–boat conformation. In the crystal structure, mol­ecules are linked into chains along the *c* axis by inter­molecular C—H⋯O and N—H⋯O hydrogen bonds. Further stabilization is provided by C—H⋯π inter­actions.

## Related literature

For applications of bicyclo­[3.3.1]nonane derivatives, see: Arias-Perez *et al.* (1997 ▶). For applications of *N*,*N*-diphenyl derivatives, see: Srikrishna & Vijayakumar (1998 ▶); Chinar Pathak *et al.* (2007 ▶). For bicyclic systems with aryl groups, see: Vijayakumar *et al.* (2000 ▶). For a related structure: see: Fun *et al.* (2009 ▶). For ring conformations, see: Cremer & Pople (1975 ▶). For bond-length data, see: Allen *et al.* (1987 ▶).
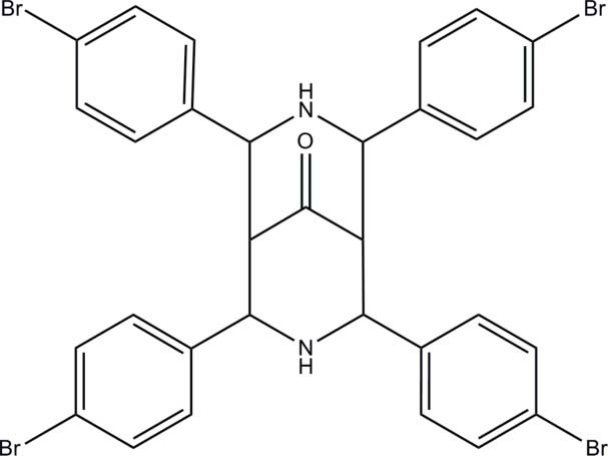


## Experimental

### 

#### Crystal data

C_31_H_24_Br_4_N_2_O
*M* *_r_* = 760.16
Monoclinic, 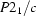

*a* = 14.7409 (5) Å
*b* = 27.7762 (10) Å
*c* = 7.1538 (2) Å
β = 101.067 (2)°
*V* = 2874.62 (16) Å^3^
*Z* = 4
Mo *K*α radiation
μ = 5.63 mm^−1^
*T* = 296 K
0.89 × 0.19 × 0.10 mm

#### Data collection

Bruker SMART APEXII CCD area-detector diffractometer
Absorption correction: multi-scan (*SADABS*; Bruker, 2009 ▶) *T*_min_ = 0.082, *T*_max_ = 0.614
37831 measured reflections
8336 independent reflections
4019 reflections with *I* > 2σ(*I*)
*R*_int_ = 0.045

#### Refinement

*R*[*F* ^2^ > 2σ(*F* ^2^)] = 0.051
*wR*(*F* ^2^) = 0.141
*S* = 1.01
8336 reflections
409 parameters
180 restraints
H-atom parameters constrained
Δρ_max_ = 0.79 e Å^−3^
Δρ_min_ = −0.79 e Å^−3^

### 

Data collection: *APEX2* (Bruker, 2009 ▶); cell refinement: *SAINT* (Bruker, 2009 ▶); data reduction: *SAINT*; program(s) used to solve structure: *SHELXTL* (Sheldrick, 2008 ▶); program(s) used to refine structure: *SHELXTL*; molecular graphics: *SHELXTL*; software used to prepare material for publication: *SHELXTL* and *PLATON* (Spek, 2009 ▶).

## Supplementary Material

Crystal structure: contains datablocks global, I. DOI: 10.1107/S1600536809055226/ci2986sup1.cif

Structure factors: contains datablocks I. DOI: 10.1107/S1600536809055226/ci2986Isup2.hkl

Additional supplementary materials:  crystallographic information; 3D view; checkCIF report

## Figures and Tables

**Table 1:** Hydrogen-bond geometry (Å, °) *Cg*1, *Cg*2 and *Cg*3 are the centroids of the C12*A*–C17*A*, C19–C24 and C26–C31 rings, respectively.

*D*—H⋯*A*	*D*—H	H⋯*A*	*D*⋯*A*	*D*—H⋯*A*
N2—H1*N*2⋯O1^i^	0.86	2.58	3.319 (4)	145
C18—H18*A*⋯O1^ii^	0.98	2.50	3.294 (5)	138
C5—H5*A*⋯*Cg*2	0.93	2.77	3.614 (5)	151
C28—H28*A*⋯*Cg*1^i^	0.93	2.67	3.433 (9)	140
C31—H31*A*⋯*Cg*3^iii^	0.93	2.80	3.640 (5)	151
C13*B*—H13*B*⋯*Cg*3	0.93	2.76	3.53 (5)	141

Symmetry codes: (i) 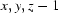; (ii) 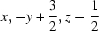; (iii) 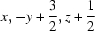.

**Fig. 1.**
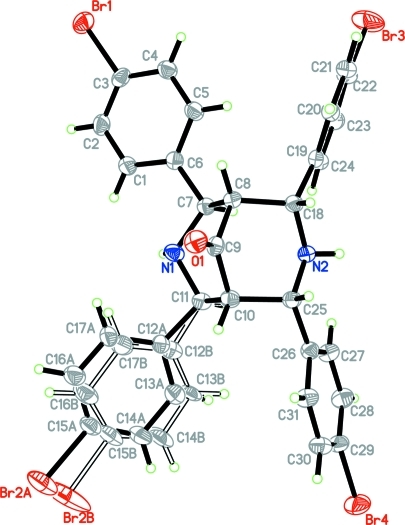
The molecular structure of the title compound, showing 30% probability displacement ellipsoids and the atom-numbering scheme. Open bonds indicate the minor disordered component.

**Fig. 2.**
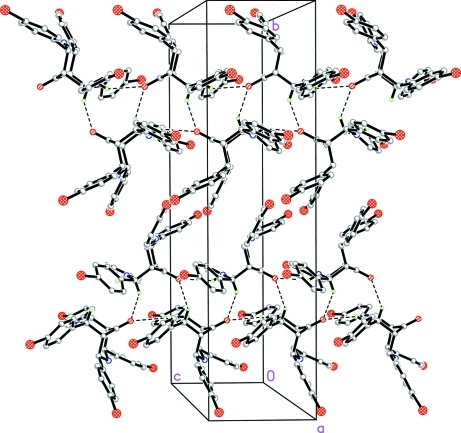
The crystal packing of the title compound, showing the extended one-dimensional chains linked along the c axis. H atoms not involved in the intermolecular interactions (dashed lines) have been omitted for clarity. Only the major components are shown.

## supplementary crystallographic information

### Comment

Bicyclo[3.3.1]nonane moieties are present in many biologically active molecules like alkaloids and drugs (Arias-Perez *et al.*, 1997). Functionalized 3-azabicyclo[3.3.1]nonanes have been studied intensively because of their pharmaceutical use and these compounds find applications as an important class of organic compounds in the field of molecular recognition. The 1,5-diphenyl-3,7-diazabicyclo[3.3.1]nonan-9-ones are local anesthetics. Some of them possess hypotensive activity. N,*N*-diphenyl derivatives are found to be antichloristic and anti-thrombic (Srikrishna & Vijayakumar, 1998; Chinar Pathak *et al.*, 2007). The synthesis and stereochemistry of 3,7-diazabicyclo[3.3.1]nonan-9-ones and their derivatives are of much interest due to their diverse biological activities, such as antibacterial, antifungal, anti-arrhythmic, antiphologistic, antithrombic, calcium antagonistic, hypotensive and neuroleptic and also because of their presence in naturally occurring lupin alkaloids. The conformational analysis of 3,7-diazabicyclo[3.3.1]nonanes (bispidines) is of considerable interest both from the theoretical view point and due to their biological activity. In recent years the 2,4,6,8-tetraaryl-3,7-diazabicyclo[3.3.1]nonanes constitutes an interesting case for the study because of the presence of four aryl groups. If all the aryls are in equatorial orientations, molecular models indicate close proximity of the aryls in both rings in the bicyclic systems (Vijayakumar *et al.*, 2000).

The bicyclo[3.3.1]nonane ring system (O1/N1/N2/C7–C11/C18/C25) adopts a chair-boat conformation with puckering parameter Q = 0.770 (4) Å, Θ = 91.8 (3)° and φ = 2.2 (3)° for one of the piperidine rings (N1/C7–C11) and Q = 0.640 (4) Å, Θ = 0.0 (4)° and φ = 139 (12)° for the other piperidine ring (N2/C8–C10/C18/C25) (Cremer & Pople, 1975). The N atoms adopt a pyramidal configuration. The phenyl rings substituted at C7 (C1–C6) and C11 [C12A–C17A (major component) and C12B–C17B (minor component)] positions are oriented with one another with an angle of 40.0 (7)° [41.2 (18)° in the minor component]. The phenyl rings substituted at C18 (C19–C24) and C25 (C26–C31) form a dihedral angle of 31.3 (2)°. Two bromophenyl groups substituted at C7 and C11 are in equatorial orientations with torsion angles C6—C7—C8—C9 = 123.3 (3)°, C9—C10—C11—C12A = -112.7 (7)° for major component and C9—C10—C11—C12B = -125.5 (19)° for minor component. The other two bromophenyl groups substituted at C18 and C25 have torsion angles of C9—C8—C18—C19 = -175.2 (3)° and C9—C10—C25—C26 = 176.6 (3)°. Bond lengths (Allen *et al.*, 1987) and angles are within the normal range and are comparable to a closely related structure (Fun *et al.*, 2009).

In the crystal structure (Fig. 2), intermolecular C18—H18A···O1 and N2—H1N2···O1 hydrogen bonds link the molecules into chains along *c* axis. The structure is further stabilized by C—H···π interactions (Table 1).

### Experimental

0.4 ml of acetone, 3.70 g of 4-bromobenzaldehyde and 0.7708 g of dry ammonium acetate were taken in a 1:4:2 molar ratio in ethanol and the mixture was heated on a water bath till it changes to red orange colour. The mixture was allowed to stand until a solid appears. The solid product was washed with ether and ethanol (1:1) until the disappearance of yellow colour. The separated solid was filtered off and recrystallized from chloroform-benzene mixture. The purity of the compound was checked by TLC and melting point recorded (yield: 57%, m. p. 511 K).

### Refinement

Atoms H1N1 and H1N2 were located in a difference Fourier map and were refined using a riding model. The remaining H hydrogen atoms were positioned geometrically [C–H = 0.93 or 0.98] and were refined using a riding model, with *U*_iso_(H) = 1.2*U*_eq_(C). One of the bromophenyl rings (C11–C17/Br2) is disordered over two positions with occupancies of 0.69 (2) and 0.31 (2). Rigid and similarity restraints were applied to the disordered ring.

### Crystal data

**Table:**

C_31_H_24_Br_4_N_2_O	*F*(000) = 1488
*M**_r_* = 760.16	*D*_x_ = 1.756 Mg m^−^^3^
Monoclinic, *P*2_1_/*c*	Mo *K*α radiation, λ = 0.71073 Å
Hall symbol: -P 2ybc	Cell parameters from 8924 reflections
*a* = 14.7409 (5) Å	θ = 2.6–25.2°
*b* = 27.7762 (10) Å	µ = 5.63 mm^−^^1^
*c* = 7.1538 (2) Å	*T* = 296 K
β = 101.067 (2)°	Plate, colourless
*V* = 2874.62 (16) Å^3^	0.89 × 0.19 × 0.10 mm
*Z* = 4	

### Data collection

**Table:**

Bruker SMART APEXII CCD area-detector diffractometer	8336 independent reflections
Radiation source: fine-focus sealed tube	4019 reflections with *I* > 2σ(*I*)
graphite	*R*_int_ = 0.045
φ and ω scans	θ_max_ = 30.1°, θ_min_ = 2.0°
Absorption correction: multi-scan (*SADABS*; Bruker, 2009)	*h* = −20→14
*T*_min_ = 0.082, *T*_max_ = 0.614	*k* = −38→31
37831 measured reflections	*l* = −10→10

### Refinement

**Table:**

Refinement on *F*^2^	Primary atom site location: structure-invariant direct methods
Least-squares matrix: full	Secondary atom site location: difference Fourier map
*R*[*F*^2^ > 2σ(*F*^2^)] = 0.051	Hydrogen site location: inferred from neighbouring sites
*wR*(*F*^2^) = 0.141	H-atom parameters constrained
*S* = 1.00	*w* = 1/[σ^2^(*F*_o_^2^) + (0.0556*P*)^2^ + 2.5808*P*] where *P* = (*F*_o_^2^ + 2*F*_c_^2^)/3
8336 reflections	(Δ/σ)_max_ = 0.001
409 parameters	Δρ_max_ = 0.79 e Å^−^^3^
180 restraints	Δρ_min_ = −0.79 e Å^−^^3^

### Special details

**Table:**

Geometry. All e.s.d.'s (except the e.s.d. in the dihedral angle between two l.s. planes) are estimated using the full covariance matrix. The cell e.s.d.'s are taken into account individually in the estimation of e.s.d.'s in distances, angles and torsion angles; correlations between e.s.d.'s in cell parameters are only used when they are defined by crystal symmetry. An approximate (isotropic) treatment of cell e.s.d.'s is used for estimating e.s.d.'s involving l.s. planes.
Refinement. Refinement of *F*^2^ against ALL reflections. The weighted *R*-factor *wR* and goodness of fit *S* are based on *F*^2^, conventional *R*-factors *R* are based on *F*, with *F* set to zero for negative *F*^2^. The threshold expression of *F*^2^ > σ(*F*^2^) is used only for calculating *R*-factors(gt) *etc*. and is not relevant to the choice of reflections for refinement. *R*-factors based on *F*^2^ are statistically about twice as large as those based on *F*, and *R*- factors based on ALL data will be even larger.

### Fractional atomic coordinates and isotropic or equivalent isotropic displacement parameters (Å^2^)

**Table:**

	*x*	*y*	*z*	*U*_iso_*/*U*_eq_	Occ. (<1)
Br1	0.31050 (3)	0.487998 (19)	0.72659 (7)	0.06406 (17)	
Br2A	1.1481 (2)	0.53133 (10)	1.3533 (4)	0.0839 (10)	0.69 (2)
Br2B	1.1745 (18)	0.5373 (5)	1.275 (5)	0.157 (9)	0.31 (2)
Br3	0.30786 (4)	0.66382 (3)	−0.10599 (9)	0.0953 (2)	
Br4	1.20123 (3)	0.67899 (2)	0.36272 (9)	0.07356 (19)	
O1	0.72350 (19)	0.69393 (11)	0.9876 (4)	0.0478 (7)	
N1	0.7408 (2)	0.58644 (12)	0.7607 (5)	0.0429 (8)	
H1N1	0.7422	0.5573	0.7230	0.036 (11)*	
N2	0.74301 (19)	0.68444 (11)	0.4561 (5)	0.0374 (7)	
H1N2	0.7425	0.7003	0.3532	0.038 (11)*	
C1	0.5800 (3)	0.53202 (15)	0.7522 (6)	0.0456 (10)	
H1A	0.6382	0.5198	0.8031	0.055*	
C2	0.5024 (3)	0.50554 (16)	0.7674 (6)	0.0492 (11)	
H2A	0.5085	0.4756	0.8265	0.059*	
C3	0.4165 (3)	0.52380 (16)	0.6947 (6)	0.0439 (10)	
C4	0.4065 (3)	0.56740 (15)	0.6027 (6)	0.0460 (10)	
H4A	0.3479	0.5793	0.5518	0.055*	
C5	0.4841 (3)	0.59328 (15)	0.5867 (6)	0.0465 (10)	
H5A	0.4773	0.6227	0.5236	0.056*	
C6	0.5728 (3)	0.57663 (14)	0.6625 (5)	0.0364 (9)	
C7	0.6570 (2)	0.60577 (13)	0.6387 (5)	0.0372 (9)	
H7A	0.6643	0.6032	0.5057	0.045*	
C8	0.6489 (2)	0.66002 (13)	0.6871 (5)	0.0345 (8)	
H8A	0.5909	0.6659	0.7313	0.041*	
C9	0.7300 (3)	0.67358 (13)	0.8402 (5)	0.0359 (9)	
C10	0.8211 (2)	0.66416 (14)	0.7791 (5)	0.0366 (9)	
H10A	0.8720	0.6733	0.8825	0.044*	
C11	0.8276 (2)	0.60953 (14)	0.7382 (6)	0.0391 (9)	
H11A	0.8388	0.6044	0.6090	0.047*	0.69 (2)
H11B	0.8321	0.6063	0.6039	0.047*	0.31 (2)
C12A	0.9042 (12)	0.5860 (11)	0.885 (3)	0.038 (3)	0.69 (2)
C13A	0.9955 (13)	0.5865 (9)	0.858 (2)	0.051 (3)	0.69 (2)
H13A	1.0078	0.5987	0.7446	0.062*	0.69 (2)
C14A	1.0693 (9)	0.5694 (6)	0.994 (2)	0.056 (3)	0.69 (2)
H14A	1.1297	0.5703	0.9737	0.068*	0.69 (2)
C15A	1.0490 (10)	0.5514 (6)	1.1589 (19)	0.052 (3)	0.69 (2)
C16A	0.9581 (9)	0.5494 (6)	1.187 (2)	0.058 (3)	0.69 (2)
H16A	0.9458	0.5360	1.2988	0.070*	0.69 (2)
C17A	0.8892 (11)	0.5664 (8)	1.058 (2)	0.048 (3)	0.69 (2)
H17A	0.8295	0.5654	1.0817	0.057*	0.69 (2)
C12B	0.915 (3)	0.590 (3)	0.853 (8)	0.047 (7)	0.31 (2)
C13B	0.995 (3)	0.5912 (19)	0.799 (5)	0.050 (6)	0.31 (2)
H13B	0.9978	0.6032	0.6788	0.060*	0.31 (2)
C14B	1.074 (2)	0.5748 (15)	0.919 (5)	0.067 (7)	0.31 (2)
H14B	1.1309	0.5748	0.8791	0.080*	0.31 (2)
C15B	1.067 (2)	0.5587 (14)	1.098 (6)	0.060 (7)	0.31 (2)
C16B	0.991 (2)	0.5515 (13)	1.157 (5)	0.066 (6)	0.31 (2)
H16B	0.9877	0.5363	1.2712	0.079*	0.31 (2)
C17B	0.905 (3)	0.571 (2)	1.018 (7)	0.061 (6)	0.31 (2)
H17B	0.8469	0.5694	1.0502	0.073*	0.31 (2)
C18	0.6555 (2)	0.69378 (14)	0.5159 (5)	0.0361 (9)	
H18A	0.6560	0.7272	0.5603	0.043*	
C19	0.5739 (2)	0.68748 (14)	0.3557 (5)	0.0354 (9)	
C20	0.4955 (3)	0.71480 (15)	0.3534 (6)	0.0425 (10)	
H20A	0.4963	0.7385	0.4455	0.051*	
C21	0.4157 (3)	0.70809 (17)	0.2188 (6)	0.0525 (11)	
H21A	0.3634	0.7267	0.2203	0.063*	
C22	0.4158 (3)	0.67304 (18)	0.0822 (6)	0.0519 (11)	
C23	0.4933 (3)	0.64617 (17)	0.0766 (6)	0.0486 (11)	
H23A	0.4927	0.6231	−0.0179	0.058*	
C24	0.5721 (3)	0.65356 (15)	0.2121 (5)	0.0417 (10)	
H24A	0.6249	0.6356	0.2074	0.050*	
C25	0.8226 (2)	0.69706 (14)	0.6039 (5)	0.0371 (9)	
H25A	0.8152	0.7305	0.6421	0.045*	
C26	0.9132 (2)	0.69287 (14)	0.5379 (5)	0.0377 (9)	
C27	0.9219 (3)	0.67096 (15)	0.3685 (6)	0.0452 (10)	
H27A	0.8697	0.6588	0.2885	0.054*	
C28	1.0073 (3)	0.66702 (16)	0.3171 (6)	0.0506 (11)	
H28A	1.0124	0.6523	0.2029	0.061*	
C29	1.0844 (3)	0.68484 (16)	0.4343 (6)	0.0479 (10)	
C30	1.0784 (3)	0.70730 (16)	0.6042 (6)	0.0505 (11)	
H30A	1.1308	0.7192	0.6844	0.061*	
C31	0.9919 (3)	0.71153 (15)	0.6511 (6)	0.0472 (10)	
H31A	0.9865	0.7275	0.7627	0.057*	

### Atomic displacement parameters (Å^2^)

**Table:**

	*U*^11^	*U*^22^	*U*^33^	*U*^12^	*U*^13^	*U*^23^
Br1	0.0530 (3)	0.0716 (4)	0.0666 (3)	−0.0215 (2)	0.0090 (2)	0.0103 (3)
Br2A	0.0692 (10)	0.0664 (8)	0.0957 (18)	0.0137 (7)	−0.0356 (12)	0.0011 (9)
Br2B	0.124 (8)	0.068 (3)	0.219 (15)	0.046 (4)	−0.118 (10)	−0.047 (6)
Br3	0.0508 (3)	0.1428 (6)	0.0794 (4)	0.0087 (3)	−0.0203 (3)	−0.0159 (4)
Br4	0.0425 (3)	0.0858 (4)	0.0980 (4)	0.0077 (2)	0.0277 (3)	0.0056 (3)
O1	0.0504 (16)	0.0562 (19)	0.0374 (16)	−0.0039 (14)	0.0100 (13)	−0.0084 (14)
N1	0.0348 (17)	0.031 (2)	0.059 (2)	0.0011 (14)	−0.0004 (15)	0.0032 (17)
N2	0.0309 (15)	0.044 (2)	0.0380 (18)	0.0006 (14)	0.0076 (13)	0.0047 (16)
C1	0.042 (2)	0.044 (3)	0.048 (2)	−0.0005 (19)	0.0045 (19)	0.008 (2)
C2	0.055 (3)	0.041 (3)	0.051 (3)	−0.008 (2)	0.008 (2)	0.011 (2)
C3	0.043 (2)	0.052 (3)	0.037 (2)	−0.0163 (19)	0.0091 (18)	−0.004 (2)
C4	0.039 (2)	0.046 (3)	0.050 (2)	−0.0014 (19)	0.0008 (18)	0.003 (2)
C5	0.041 (2)	0.042 (3)	0.053 (3)	−0.0031 (19)	−0.0015 (19)	0.007 (2)
C6	0.039 (2)	0.036 (2)	0.033 (2)	−0.0031 (16)	0.0044 (16)	−0.0024 (17)
C7	0.0338 (19)	0.038 (2)	0.039 (2)	−0.0013 (16)	0.0048 (16)	0.0010 (18)
C8	0.0306 (18)	0.037 (2)	0.037 (2)	−0.0005 (16)	0.0084 (15)	−0.0014 (17)
C9	0.043 (2)	0.031 (2)	0.036 (2)	−0.0008 (17)	0.0112 (17)	0.0006 (18)
C10	0.0330 (18)	0.040 (2)	0.035 (2)	−0.0005 (16)	0.0041 (15)	−0.0048 (17)
C11	0.0332 (19)	0.041 (2)	0.041 (2)	0.0009 (17)	0.0029 (17)	−0.0018 (18)
C12A	0.039 (5)	0.022 (5)	0.052 (7)	0.000 (4)	0.004 (4)	0.006 (5)
C13A	0.048 (5)	0.052 (7)	0.053 (7)	0.007 (4)	0.006 (5)	0.007 (6)
C14A	0.041 (4)	0.067 (9)	0.057 (9)	0.012 (4)	−0.003 (6)	0.004 (8)
C15A	0.041 (5)	0.040 (6)	0.065 (7)	0.002 (4)	−0.012 (5)	0.012 (5)
C16A	0.046 (6)	0.049 (5)	0.072 (6)	−0.007 (5)	−0.006 (4)	0.014 (4)
C17A	0.046 (5)	0.045 (6)	0.049 (6)	−0.003 (4)	0.001 (4)	0.016 (4)
C12B	0.040 (9)	0.040 (16)	0.057 (14)	0.002 (11)	−0.004 (10)	−0.004 (11)
C13B	0.029 (7)	0.044 (11)	0.069 (15)	0.010 (7)	−0.011 (10)	0.011 (14)
C14B	0.049 (9)	0.057 (12)	0.083 (17)	0.022 (9)	−0.018 (11)	0.012 (15)
C15B	0.051 (10)	0.041 (14)	0.073 (16)	0.008 (10)	−0.028 (11)	0.002 (14)
C16B	0.059 (13)	0.054 (11)	0.070 (12)	−0.009 (14)	−0.027 (10)	0.003 (9)
C17B	0.045 (10)	0.060 (14)	0.071 (15)	0.000 (10)	−0.003 (9)	0.011 (13)
C18	0.0325 (18)	0.029 (2)	0.046 (2)	0.0024 (15)	0.0076 (17)	0.0019 (18)
C19	0.0350 (19)	0.036 (2)	0.037 (2)	0.0005 (16)	0.0091 (16)	0.0103 (18)
C20	0.041 (2)	0.045 (3)	0.043 (2)	0.0067 (18)	0.0115 (18)	0.0083 (19)
C21	0.036 (2)	0.065 (3)	0.057 (3)	0.008 (2)	0.011 (2)	0.015 (3)
C22	0.032 (2)	0.077 (3)	0.044 (2)	−0.001 (2)	−0.0004 (17)	0.008 (2)
C23	0.044 (2)	0.066 (3)	0.035 (2)	0.001 (2)	0.0071 (18)	0.000 (2)
C24	0.037 (2)	0.049 (3)	0.040 (2)	0.0068 (18)	0.0106 (17)	0.007 (2)
C25	0.0312 (18)	0.037 (2)	0.043 (2)	0.0000 (16)	0.0067 (16)	0.0007 (18)
C26	0.0347 (19)	0.035 (2)	0.043 (2)	0.0007 (16)	0.0067 (17)	0.0014 (18)
C27	0.040 (2)	0.049 (3)	0.048 (2)	−0.0043 (19)	0.0114 (18)	−0.006 (2)
C28	0.048 (2)	0.055 (3)	0.052 (3)	0.001 (2)	0.018 (2)	−0.009 (2)
C29	0.032 (2)	0.049 (3)	0.064 (3)	0.0026 (18)	0.0131 (19)	0.007 (2)
C30	0.035 (2)	0.057 (3)	0.056 (3)	−0.004 (2)	0.0005 (19)	−0.004 (2)
C31	0.040 (2)	0.051 (3)	0.051 (2)	−0.0043 (19)	0.0090 (19)	−0.008 (2)

### Geometric parameters (Å, °)

**Table:**

Br1—C3	1.902 (4)	C14A—C15A	1.365 (12)
Br2A—C15A	1.897 (13)	C14A—H14A	0.93
Br2B—C15B	1.92 (3)	C15A—C16A	1.394 (13)
Br3—C22	1.894 (4)	C16A—C17A	1.32 (2)
Br4—C29	1.895 (4)	C16A—H16A	0.93
O1—C9	1.217 (4)	C17A—H17A	0.93
N1—C11	1.468 (5)	C12B—C13B	1.30 (5)
N1—C7	1.470 (5)	C12B—C17B	1.32 (4)
N1—H1N1	0.85	C13B—C14B	1.39 (3)
N2—C18	1.458 (4)	C13B—H13B	0.93
N2—C25	1.463 (5)	C14B—C15B	1.38 (4)
N2—H1N2	0.86	C14B—H14B	0.93
C1—C2	1.381 (6)	C15B—C16B	1.29 (4)
C1—C6	1.390 (5)	C16B—C17B	1.55 (5)
C1—H1A	0.93	C16B—H16B	0.93
C2—C3	1.371 (6)	C17B—H17B	0.93
C2—H2A	0.93	C18—C19	1.504 (5)
C3—C4	1.373 (6)	C18—H18A	0.98
C4—C5	1.375 (5)	C19—C20	1.380 (5)
C4—H4A	0.93	C19—C24	1.391 (5)
C5—C6	1.394 (5)	C20—C21	1.382 (5)
C5—H5A	0.93	C20—H20A	0.93
C6—C7	1.519 (5)	C21—C22	1.379 (6)
C7—C8	1.556 (5)	C21—H21A	0.93
C7—H7A	0.98	C22—C23	1.373 (6)
C8—C9	1.506 (5)	C23—C24	1.377 (5)
C8—C18	1.560 (5)	C23—H23A	0.93
C8—H8A	0.98	C24—H24A	0.93
C9—C10	1.513 (5)	C25—C26	1.504 (5)
C10—C11	1.552 (5)	C25—H25A	0.98
C10—C25	1.555 (5)	C26—C31	1.382 (5)
C10—H10A	0.98	C26—C27	1.383 (5)
C11—C12B	1.50 (4)	C27—C28	1.382 (5)
C11—C12A	1.533 (19)	C27—H27A	0.93
C11—H11A	0.98	C28—C29	1.369 (6)
C11—H11B	0.98	C28—H28A	0.93
C12A—C13A	1.40 (2)	C29—C30	1.384 (6)
C12A—C17A	1.406 (15)	C30—C31	1.384 (5)
C13A—C14A	1.396 (16)	C30—H30A	0.93
C13A—H13A	0.93	C31—H31A	0.93
			
C11—N1—C7	115.2 (3)	C17A—C16A—H16A	119.5
C11—N1—H1N1	107.7	C15A—C16A—H16A	119.5
C7—N1—H1N1	103.1	C16A—C17A—C12A	121.6 (12)
C18—N2—C25	112.2 (3)	C16A—C17A—H17A	119.2
C18—N2—H1N2	107.3	C12A—C17A—H17A	119.2
C25—N2—H1N2	111.8	C13B—C12B—C17B	123 (4)
C2—C1—C6	121.3 (4)	C13B—C12B—C11	124 (3)
C2—C1—H1A	119.3	C17B—C12B—C11	114 (4)
C6—C1—H1A	119.3	C12B—C13B—C14B	120 (3)
C3—C2—C1	119.5 (4)	C12B—C13B—H13B	119.8
C3—C2—H2A	120.3	C14B—C13B—H13B	119.8
C1—C2—H2A	120.3	C15B—C14B—C13B	118 (3)
C2—C3—C4	121.0 (4)	C15B—C14B—H14B	120.9
C2—C3—Br1	118.8 (3)	C13B—C14B—H14B	121.0
C4—C3—Br1	120.2 (3)	C16B—C15B—C14B	125 (3)
C3—C4—C5	119.1 (4)	C16B—C15B—Br2B	113 (3)
C3—C4—H4A	120.5	C14B—C15B—Br2B	121 (3)
C5—C4—H4A	120.5	C15B—C16B—C17B	113 (2)
C4—C5—C6	121.8 (4)	C15B—C16B—H16B	123.4
C4—C5—H5A	119.1	C17B—C16B—H16B	123.4
C6—C5—H5A	119.1	C12B—C17B—C16B	119 (3)
C1—C6—C5	117.3 (4)	C12B—C17B—H17B	120.3
C1—C6—C7	122.3 (3)	C16B—C17B—H17B	120.3
C5—C6—C7	120.3 (3)	N2—C18—C19	112.3 (3)
N1—C7—C6	110.5 (3)	N2—C18—C8	108.4 (3)
N1—C7—C8	108.3 (3)	C19—C18—C8	111.5 (3)
C6—C7—C8	113.0 (3)	N2—C18—H18A	108.2
N1—C7—H7A	108.3	C19—C18—H18A	108.2
C6—C7—H7A	108.3	C8—C18—H18A	108.2
C8—C7—H7A	108.3	C20—C19—C24	117.8 (4)
C9—C8—C7	108.6 (3)	C20—C19—C18	119.4 (4)
C9—C8—C18	105.0 (3)	C24—C19—C18	122.7 (3)
C7—C8—C18	112.8 (3)	C19—C20—C21	122.2 (4)
C9—C8—H8A	110.1	C19—C20—H20A	118.9
C7—C8—H8A	110.1	C21—C20—H20A	118.9
C18—C8—H8A	110.1	C22—C21—C20	118.2 (4)
O1—C9—C8	124.3 (3)	C22—C21—H21A	120.9
O1—C9—C10	123.6 (3)	C20—C21—H21A	120.9
C8—C9—C10	111.8 (3)	C23—C22—C21	121.2 (4)
C9—C10—C11	108.3 (3)	C23—C22—Br3	119.8 (4)
C9—C10—C25	106.3 (3)	C21—C22—Br3	119.0 (3)
C11—C10—C25	114.3 (3)	C22—C23—C24	119.6 (4)
C9—C10—H10A	109.3	C22—C23—H23A	120.2
C11—C10—H10A	109.3	C24—C23—H23A	120.2
C25—C10—H10A	109.3	C23—C24—C19	120.9 (4)
N1—C11—C12B	117 (2)	C23—C24—H24A	119.5
N1—C11—C12A	106.3 (8)	C19—C24—H24A	119.5
N1—C11—C10	108.7 (3)	N2—C25—C26	113.1 (3)
C12B—C11—C10	109 (3)	N2—C25—C10	108.0 (3)
C12A—C11—C10	110.6 (13)	C26—C25—C10	111.0 (3)
N1—C11—H11A	110.4	N2—C25—H25A	108.2
C12B—C11—H11A	100.5	C26—C25—H25A	108.2
C12A—C11—H11A	110.4	C10—C25—H25A	108.2
C10—C11—H11A	110.4	C31—C26—C27	118.0 (4)
N1—C11—H11B	107.0	C31—C26—C25	118.9 (3)
C12B—C11—H11B	107.0	C27—C26—C25	123.1 (3)
C12A—C11—H11B	116.9	C28—C27—C26	120.7 (4)
C10—C11—H11B	107.0	C28—C27—H27A	119.7
C13A—C12A—C17A	116.3 (14)	C26—C27—H27A	119.7
C13A—C12A—C11	120.3 (12)	C29—C28—C27	120.0 (4)
C17A—C12A—C11	123.2 (13)	C29—C28—H28A	120.0
C12A—C13A—C14A	122.9 (10)	C27—C28—H28A	120.0
C12A—C13A—H13A	118.6	C28—C29—C30	120.9 (4)
C14A—C13A—H13A	118.6	C28—C29—Br4	119.6 (3)
C15A—C14A—C13A	117.1 (10)	C30—C29—Br4	119.4 (3)
C15A—C14A—H14A	121.4	C31—C30—C29	118.0 (4)
C13A—C14A—H14A	121.4	C31—C30—H30A	121.0
C14A—C15A—C16A	121.1 (10)	C29—C30—H30A	121.0
C14A—C15A—Br2A	118.3 (10)	C26—C31—C30	122.3 (4)
C16A—C15A—Br2A	120.5 (8)	C26—C31—H31A	118.8
C17A—C16A—C15A	120.9 (10)	C30—C31—H31A	118.8
			
C6—C1—C2—C3	−0.8 (6)	C10—C11—C12B—C13B	−85 (6)
C1—C2—C3—C4	1.7 (6)	N1—C11—C12B—C17B	−30 (7)
C1—C2—C3—Br1	−177.4 (3)	C12A—C11—C12B—C17B	−4(16)
C2—C3—C4—C5	−1.1 (6)	C10—C11—C12B—C17B	94 (6)
Br1—C3—C4—C5	178.0 (3)	C17B—C12B—C13B—C14B	−4(9)
C3—C4—C5—C6	−0.5 (6)	C11—C12B—C13B—C14B	176 (5)
C2—C1—C6—C5	−0.7 (6)	C12B—C13B—C14B—C15B	−2(7)
C2—C1—C6—C7	−178.1 (4)	C13B—C14B—C15B—C16B	10 (7)
C4—C5—C6—C1	1.3 (6)	C13B—C14B—C15B—Br2B	−178 (3)
C4—C5—C6—C7	178.8 (4)	C14B—C15B—C16B—C17B	−10 (6)
C11—N1—C7—C6	175.4 (3)	Br2B—C15B—C16B—C17B	177 (3)
C11—N1—C7—C8	−60.3 (4)	C13B—C12B—C17B—C16B	3(10)
C1—C6—C7—N1	−13.9 (5)	C11—C12B—C17B—C16B	−177 (4)
C5—C6—C7—N1	168.8 (3)	C15B—C16B—C17B—C12B	4(7)
C1—C6—C7—C8	−135.4 (4)	C25—N2—C18—C19	172.0 (3)
C5—C6—C7—C8	47.3 (5)	C25—N2—C18—C8	−64.4 (4)
N1—C7—C8—C9	0.5 (4)	C9—C8—C18—N2	60.7 (4)
C6—C7—C8—C9	123.3 (3)	C7—C8—C18—N2	−57.4 (4)
N1—C7—C8—C18	116.6 (3)	C9—C8—C18—C19	−175.2 (3)
C6—C7—C8—C18	−120.6 (3)	C7—C8—C18—C19	66.7 (4)
C7—C8—C9—O1	−128.3 (4)	N2—C18—C19—C20	−149.5 (3)
C18—C8—C9—O1	110.7 (4)	C8—C18—C19—C20	88.7 (4)
C7—C8—C9—C10	58.4 (4)	N2—C18—C19—C24	33.4 (5)
C18—C8—C9—C10	−62.6 (4)	C8—C18—C19—C24	−88.5 (4)
O1—C9—C10—C11	125.8 (4)	C24—C19—C20—C21	2.5 (6)
C8—C9—C10—C11	−60.8 (4)	C18—C19—C20—C21	−174.8 (4)
O1—C9—C10—C25	−110.9 (4)	C19—C20—C21—C22	−0.5 (6)
C8—C9—C10—C25	62.4 (4)	C20—C21—C22—C23	−1.4 (7)
C7—N1—C11—C12B	−178 (3)	C20—C21—C22—Br3	−179.1 (3)
C7—N1—C11—C12A	176.9 (13)	C21—C22—C23—C24	1.3 (7)
C7—N1—C11—C10	57.8 (4)	Br3—C22—C23—C24	179.0 (3)
C9—C10—C11—N1	3.6 (4)	C22—C23—C24—C19	0.7 (6)
C25—C10—C11—N1	−114.7 (3)	C20—C19—C24—C23	−2.6 (6)
C9—C10—C11—C12B	−125.5 (19)	C18—C19—C24—C23	174.6 (4)
C25—C10—C11—C12B	116.2 (19)	C18—N2—C25—C26	−173.7 (3)
C9—C10—C11—C12A	−112.7 (7)	C18—N2—C25—C10	63.1 (4)
C25—C10—C11—C12A	128.9 (7)	C9—C10—C25—N2	−59.0 (4)
N1—C11—C12A—C13A	157 (2)	C11—C10—C25—N2	60.4 (4)
C12B—C11—C12A—C13A	1(19)	C9—C10—C25—C26	176.6 (3)
C10—C11—C12A—C13A	−85 (2)	C11—C10—C25—C26	−64.0 (4)
N1—C11—C12A—C17A	−28 (3)	N2—C25—C26—C31	168.9 (4)
C12B—C11—C12A—C17A	176 (23)	C10—C25—C26—C31	−69.6 (5)
C10—C11—C12A—C17A	89 (3)	N2—C25—C26—C27	−11.3 (5)
C17A—C12A—C13A—C14A	−1(4)	C10—C25—C26—C27	110.2 (4)
C11—C12A—C13A—C14A	174 (2)	C31—C26—C27—C28	1.5 (6)
C12A—C13A—C14A—C15A	0(3)	C25—C26—C27—C28	−178.3 (4)
C13A—C14A—C15A—C16A	1(2)	C26—C27—C28—C29	0.1 (7)
C13A—C14A—C15A—Br2A	−176.4 (14)	C27—C28—C29—C30	−0.5 (7)
C14A—C15A—C16A—C17A	−2(3)	C27—C28—C29—Br4	179.8 (3)
Br2A—C15A—C16A—C17A	175.3 (15)	C28—C29—C30—C31	−0.6 (7)
C15A—C16A—C17A—C12A	2(3)	Br4—C29—C30—C31	179.2 (3)
C13A—C12A—C17A—C16A	0(4)	C27—C26—C31—C30	−2.6 (6)
C11—C12A—C17A—C16A	−175 (2)	C25—C26—C31—C30	177.2 (4)
N1—C11—C12B—C13B	150 (5)	C29—C30—C31—C26	2.2 (7)
C12A—C11—C12B—C13B	177 (26)		

### Hydrogen-bond geometry (Å, °)

**Table:**

Cg1, Cg2 and Cg3 are the centroids of the C12A–C17A, C19–C24 and C26–C31 rings, respectively.

**Table:**

*D*—H···*A*	*D*—H	H···*A*	*D*···*A*	*D*—H···*A*
N2—H1N2···O1^i^	0.86	2.58	3.319 (4)	145
C18—H18A···O1^ii^	0.98	2.50	3.294 (5)	138
C5—H5A···Cg2	0.93	2.77	3.614 (5)	151
C28—H28A···Cg1^i^	0.93	2.67	3.433 (9)	140
C31—H31A···Cg3^iii^	0.93	2.80	3.640 (5)	151
C13B—H13B···Cg3	0.93	2.76	3.53 (5)	141

Symmetry codes: (i) *x*, *y*, *z*−1; (ii) *x*, −*y*+3/2, *z*−1/2;  (iii) *x*, −*y*+3/2, *z*+1/2.
